# Deep learning based classification of dynamic processes in time-resolved X-ray tomographic microscopy

**DOI:** 10.1038/s41598-021-03546-8

**Published:** 2021-12-17

**Authors:** Minna Bührer, Hong Xu, Allard A. Hendriksen, Felix N. Büchi, Jens Eller, Marco Stampanoni, Federica Marone

**Affiliations:** 1 Swiss Light Source, Paul Scherrer Institut, Forschungsstrasse 111, 5232 Villigen, Aargau Switzerland; 2 Institute for Biomedical Engineering, University and ETH Zürich, 8092 Zurich, Zürich Switzerland; 3 Electrochemistry Laboratory, Paul Scherrer Institut, Forschungsstrasse 111, 5232 Villigen, Aargau Switzerland; 4 Centrum Wiskunde & Informatica, Science Park 123, 1098 XG Amsterdam, The Netherlands

**Keywords:** Fuel cells, Imaging techniques, Microscopy, Computational science, Scientific data, Software

## Abstract

Time-resolved X-ray tomographic microscopy is an invaluable technique to investigate dynamic processes in 3D for extended time periods. Because of the limited signal-to-noise ratio caused by the short exposure times and sparse angular sampling frequency, obtaining quantitative information through post-processing remains challenging and requires intensive manual labor. This severely limits the accessible experimental parameter space and so, prevents fully exploiting the capabilities of the dedicated time-resolved X-ray tomographic stations. Though automatic approaches, often exploiting iterative reconstruction methods, are currently being developed, the required computational costs typically remain high. Here, we propose a highly efficient reconstruction and classification pipeline (SIRT-FBP-MS-D-DIFF) that combines an algebraic filter approximation and machine learning to significantly reduce the computational time. The dynamic features are reconstructed by standard filtered back-projection with an algebraic filter to approximate iterative reconstruction quality in a computationally efficient manner. The raw reconstructions are post-processed with a trained convolutional neural network to extract the dynamic features from the low signal-to-noise ratio reconstructions in a fully automatic manner. The capabilities of the proposed pipeline are demonstrated on three different dynamic fuel cell datasets, one exploited for training and two for testing without network retraining. The proposed approach enables automatic processing of several hundreds of datasets in a single day on a single GPU node readily available at most institutions, so extending the possibilities in future dynamic X-ray tomographic investigations.

## Introduction

X-ray tomography is a widespread imaging technique used to investigate the internal structures of a large variety of opaque samples in a non-destructive manner. During the past decade, dynamic tomographic microscopy has paved the way towards the volumetric investigation of time-evolving processes, as for instance crack formation during in situ tensile tests^1^ and metal foaming^2^. In dynamic tomographic investigations the speed of evolving non-periodic processes dictates the imaging conditions, and the maximum allowed scan time, to avoid movement artefacts. Fast dynamic processes with characteristic time-scales in the sub-second range typically strongly limit the possible exposure time and angular sampling frequency, usually leading to highly noisy reconstructions also affected by undersampling artefacts. Prior to quantitative analysis, a series of dedicated, labor-intensive and time-consuming post-processing steps (such as registration, filtering and segmentation) is therefore often needed. Parameter tuning and manual optimization are mostly unavoidable: the total data post-processing time is therefore significantly dominated by the human component and not by the actual computation time. Furthermore parameter optimization is unfortunately often dataset specific. The analysis of tens of TBs of data from multiple samples and/or different experimental conditions is not realistic. While iterative reconstruction algorithms (e.g.^3–11^) can be exploited to enhance the raw reconstruction quality and ease the following data post-processing task, they often also require parameter tuning through a time-consuming trial-and-error process and are typically computationally intensive. Daily experience at dedicated time-resolved X-ray tomographic stations shows that unfortunately their capabilities are often not fully exploited: experimental plans are mostly limited to ensure feasible data processing or the data analysis is restricted to only a part of the acquired data.

Sub-optimal water management during the operation of polymer electrolyte fuel cells (PEFCs), a promising technology considered in future cleaner energy strategies, limits their performance maximization towards higher power densities. Dynamic sub-second X-ray tomographic microscopy has proven pivotal in the investigation of liquid water dynamics in fuel cells during transient operation^12–15^. To reach the required sub-second time resolution, both angular sampling frequency and exposure time are minimized leading to noisy datasets, which after tomographic reconstruction with standard analytical reconstruction algorithms result in low signal-to-noise ratio (SNR) volumes with undersampling artifacts^13^. Moreover, the reconstructed liquid water, static structures and noise have similar gray level values, hindering the classification of water features directly from the reconstructed dynamic volumes. To overcome this challenge, a high-quality *post operando* tomographic volume of the fuel cell in a dry state is registered to and subtracted from each reconstructed dynamic time frame to obtain difference image volumes containing only liquid water features, background noise and misalignment artifacts due to insufficient alignment of the static and dynamic reconstructed datasets. A dedicated ad hoc pipeline^13,16^ is then used to ensure that only the correct features are segmented as water. The cell components and the imaging protocols typically vary between different experiments addressing different scientific questions. The parameters of the post-processing pipeline are material and experiment dependent and need to be individually and often manually adjusted hindering any automation and as a consequence efficient quantitative analysis for larger comprehensive studies.

In 2020, Bührer et al.^17^ proposed an iterative reconstruction algorithm to automate the reconstruction and feature extraction of dynamic components from static matrices. The performance of the proposed algorithm was explored on both simulated and real fuel cell tomography datasets, yielding high accuracy when compared to manual segmentation. The proposed approach enabled the full automation of the reconstruction and segmentation pipeline, completely decoupling the data processing from manual efforts. Compared to the manual segmentation pipeline used thus far^13^, for which the processing time is strongly dominated by manual work, the proposed automatic pipeline enabled at least a factor of 4 reduction in the processing time while being fully automatic. However, the computational time remained still relatively high on limited computational resources.

In recent years, machine learning has demonstrated applicability to a wide range of imaging problems. Convolutional neural networks (CNNs) in particular have been applied successfully to various image processing tasks such as image classification (e.g.^18,19^), semantic segmentation (e.g.^20,21^) and image enhancement (e.g.^22–26^). In computed tomography (CT), a variety of CNN models have been successfully applied as a post-processing tool to remove reconstruction artefacts and noise from reconstructions obtained through classical reconstruction methods^19,24,27–30^. In addition, neural networks have also been exploited to learn a filter bank for the FBP reconstruction method to directly improve the reconstruction quality within the reconstruction algorithm^31^. CNNs have also demonstrated capabilities as an end-to-end model that learns the mapping between the sinogram and reconstructed image based on training data^32^. In such an end-to-end model the geometry of the measurement process is though completely ignored while Bazrafkan et al. demonstrated that for tomographic images the auxiliary information associated with the scanning geometry can help network models to retain more detailed structures^33^. Though training of CNNs can be relatively time-consuming, the trained networks can be applied efficiently even to image sizes typical in synchrotron tomography experiments: as an example, a 100-layer mixed-scale dense convolutional network (MS-D) has demonstrated capabilities to process a single reconstructed image of 1024 × 1024 pixels in only 200 ms on an Nvidia GTX 1080 GPU^24^. The trained networks can often be directly applied to other similar datasets that are collected under similar experimental conditions. Completely different sample structures, compositions and experimental conditions typically require retraining of the network. For dynamic tomographic experiments where several different samples are imaged under a variety of experimental parameters, a reality in fuel cell experiments, the required retraining of the related large data volumes significantly extends the complete post-processing time. Moreover, as it is often difficult to obtain high-quality training data, especially for semantic segmentation of low SNR datasets, applicability of deep learning approaches to such dynamic investigations still remains a great challenge.

In this paper, we propose a highly-efficient algorithm protocol that combines deep learning and algebraic filters to tomographically reconstruct and segment dynamic features in large data volumes acquired in time-resolved experiments. The proposed algorithm follows the difference sinogram approach proposed by Bührer et al.^17^ to separate the dynamic features from the static matrix prior reconstruction to minimize any potential misalignment artefacts in a fully automatic manner. The difference sinograms are then reconstructed through the SIRT-FBP algorithm^34^, which approximates the simultaneous iterative reconstruction technique (SIRT) through an angle-dependent filter within the standard FBP algorithm. This enables reconstructing the raw data volumes in the same computational time as standard FBP with the reconstruction quality of standard SIRT. The reconstruction step is automatically followed by the extraction of the dynamic features from the noisy difference reconstructions through trained deep convolutional neural networks. This enables to efficiently reconstruct and segment the dynamic features from the collected data volumes, allowing to process hundreds of datasets in a single day on a single GPU node. Moreover, as the network is trained on difference reconstructions while the complex static structures are completely ignored, the trained network can be applied to additional datasets with different static structures without retraining, so significantly reducing the total computational burden. The proposed algorithm performance is demonstrated on three real-world fuel cell datasets, however, it can be applied to any dataset where evolving dynamics are present.

## Methods

### Background

#### Tomography model

The tomography model is defined for a 2D parallel beam geometry experiment by the linear system ${\varvec{p}}={\varvec{W}}{\varvec{x}}.$ The column vector ${\varvec{x}}=\left({x}_{j}\right) \epsilon {\mathbb{R}}^{N}$ represents the unknown object, defined on a grid of $N$ pixels. The vector ${\varvec{p}}=\left({p}_{i}\right)\in {\mathbb{R}}^{M}$ consists of log-corrected measured or phase-retrieved projection values of the object ${\varvec{x}}$ from all angular positions, typically distributed homogeneously between 0° and 180° in parallel-beam geometry. ${\varvec{W}}=\left({w}_{ij}\right)\in {\mathbb{R}}^{M\times N}$ is a collection of weights that model the contribution of each pixel $j$ to the projected value at index $i$.

In tomographic reconstruction the aim is to reconstruct the unknown object ${\varvec{x}}$ from its measurement ${\varvec{p}}$. A direct solution of the system of linear equations is typically impracticable due to its ill-posedness. The reconstruction accuracy with analytical reconstruction methods, such as filtered back-projection (FBP)^35^, is high, if a sufficient amount of homogenously distributed angular views of the object are available. If the number of available angular views is insufficient, the angular sampling is highly sparse or the measurement angle range is restricted (limited-angle tomography), the quality of tomographic volumes obtained with analytical reconstruction algorithms is deteriorated by streak artefacts. Iterative reconstruction algorithms deal with these limitations by modeling the measurement through a linear system of equations and exploiting prior information of the object within the reconstruction algorithm.

#### Conventional FBP and SIRT

A common analytical reconstruction algorithm for solving the tomographic inverse problem is FBP^35^. In FBP, the measured projection data ${\varvec{p}}$ is convolved with a filter ${\varvec{h}}\boldsymbol{ }\in \boldsymbol{ }{\mathbb{R}}^{{\varvec{M}}}$ prior to the back-projection step, defined^34,36^ by

1 $$\mathrm{FBP}\left({\varvec{p}},{\varvec{h}}\right)={{\varvec{W}}}^{T}{{\varvec{C}}}_{{\varvec{h}}}{\varvec{p}},$$

where ${{\varvec{C}}}_{{\varvec{h}}}{\varvec{p}}$ denotes a convolution of the filter ${\varvec{h}}$ with the measured data ${\varvec{p}}$, and ${{\varvec{W}}}^{T}$ is the back-projection operator. The filter ${\varvec{h}}$ can be chosen depending on the SNR of the measured data to optimize the spatial resolution and contrast of the reconstruction.

The Simultaneous Iterative Reconstruction Technique (SIRT)^37,38^ is an algebraic iterative reconstruction algorithm that models the tomographic linear system of equations. Starting from an initial reconstruction ${{\varvec{x}}}^{(0)}$, typically a zero vector, the SIRT algorithm updates the reconstruction at each iteration $k$ by

2 $${{\varvec{x}}}^{\left(k\right)}= {{\varvec{x}}}^{(k-1)}+\alpha {{\varvec{W}}}^{T}\left({\varvec{p}}-{\varvec{W}}{{\varvec{x}}}^{\left(k-1\right)}\right),$$

where $\alpha$ is a relaxation factor influencing the convergence rate^36^. The algorithm is known to converge to a solution of

3 $${\mathrm{argmin}}_{x }\left({\parallel {\varvec{W}}{\varvec{x}}-{\varvec{p}}\parallel }_{2}^{2}\right).$$

#### SIRT-FBP

In 2015, Pelt and Batenburg^34^ proposed the SIRT-FBP reconstruction method designed to accurately approximate SIRT reconstructions with the computational efficiency of standard FBP. The proposed method differs from the conventional FBP only by its filtering step, making it possible to combine the filter with the already existing, computationally efficient FBP reconstructions. In^34^ it is shown that the SIRT-FBP reconstruction corresponding to the standard SIRT reconstruction ${{\varvec{x}}}^{\left(k\right)}$ at iteration $k$ is defined by

4 $${{\varvec{x}}}^{\left(k\right)} \approx \mathrm{FBP}\left(\mathbf{p}, {{\varvec{u}}}_{k}\right)= {{\varvec{W}}}^{T}{{\varvec{C}}}_{{{\varvec{u}}}_{k}}{\varvec{p}}.$$

The angle-dependent filter ${{\varvec{u}}}_{k}$ can be computed for any number of projections, angular range and number of standard SIRT iterations. The filter computation process has a comparable computation time to a single SIRT reconstruction with the same number of iterations. Once the filter is calculated, the same filter can be though applied to any other dataset that has the same number of projections, angular range and desired number of SIRT iterations in a computation time of a standard FBP reconstruction. In 2017, Pelt and Andrade^36^ further demonstrated successful application of the algebraic filter approximation approach to several real-world large tomographic datasets, combining the filter both with FBP and Gridrec algorithms. A more detailed mathematical derivation of the filter and its performance characterization are presented in^34,36^.

#### Mixed-scale dense convolutional neural network

Machine learning models, convolutional neural networks (CNNs) in particular, have recently become popular post-processing techniques to both improve reconstruction image quality and segment the reconstructed data volumes. In typical CNNs, images are processed in successive layers, each layer consisting of multiple images. At each layer, images are computed by using learned filters to convolve the images received from the previous layers and by applying a non-linear activation function to each pixel of the convolved images. Considering ${n}_{i}$ images at the layer $i$ of the network model, the image $j$ can be described by ${z}_{i}^{j}\in {\mathbb{R}}^{N}$, computed by

5 $${z}_{i}^{j}= \sigma \left(\sum_{k=0}^{{n}_{i-1}-1}{{\varvec{H}}}_{{{\varvec{q}}}_{ijk}}{z}_{i-1}^{k}+{b}_{ij}\right),$$

where ${{\varvec{H}}}_{{{\varvec{q}}}_{ijk}}$ is a 2D convolution with a learned filter ${{\varvec{q}}}_{ijk}\in {\mathbb{R}}^{3\times 3}$, ${b}_{ij}\in {\mathbb{R}}$ is a learned bias of each layer image and $\sigma \in {\mathbb{R}}^{N}\to {\mathbb{R}}^{N}$ is a non-linear pixel-wise activation function, e.g., the ReLU function^39^. At the final layer stage, the activation function is typically different, the exact function depending on the image processing task, e.g. soft-max for segmentation or identity for image enhancement. For CNNs, the network parameters are usually learned during the training process by presenting to the network a large set of input images and corresponding target “ground truth” images.

In 2017, Pelt and Sethian^19^ proposed a mixed-scale dense (MS-D) convolutional neural network architecture design to overcome the challenge of learning a large number (typically several millions) of parameters and the requirement for a large amount of training images and computer memory related to popular encoder-decoder architectures such as U-Net^20^ when applied to large datasets. In the MS-D architecture features are captured at various image scales by using dilated convolutions^40^ with channel-specific dilation ${d}_{i}\in {\mathbb{Z}}^{+}$ in channel $i$. As all feature maps are densely connected, each layer of the network is able to directly leverage the features extracted at all intermediate layers. This architecture design enables a substantial reduction of the number of training parameters, thereby reducing the overfitting risk, the number of necessary training image pairs and computer memory requirements. As the network learns during training which combinations of dilations to use for each given training task, it adapts automatically and can therefore be easily applied to a variety of problems without architectural changes or time-consuming hyperparameter tuning. The MS-D architecture has demonstrated accurate results with relatively few intermediate images and trainable parameters^19,21,23,24^, allowing effective training with relatively small training sets and large images, also for image enhancement and segmentation tasks^19^. In Pelt et al*.*^24^ the network architecture has for instance been successfully applied to simulated and real-world tomographic data as a post-processing operator, demonstrating significant improvement in obtained reconstructed image quality for datasets suffering from different data limitations such as limited angular range and low SNR. As MS-D networks use the same set of standard machine learning operations at each layer, they are easy to implement, train, apply and modify allowing their flexible use in practice. A more detailed description of the MS-D network architecture and its performance characterization are presented in^19^.

### SIRT-FBP-MS-D-DIFF

Here, we propose an algorithm protocol designed to efficiently reconstruct and segment large quantities of low SNR data produced by dynamic experiments on moderate computational resources. The algorithm consists of a data reconstruction part (SIRT-FBP) and a feature extraction part (MS-D), which together provide an automatic and efficient reconstruction and segmentation pipeline for large quantities of low SNR tomographic data.

#### Data reconstruction

The data reconstruction step follows closely the protocol proposed by Bührer et al.^17^ For samples where dynamics are present within a static matrix, an additional tomographic scan of the sample in a static state (without dynamics) is required to apply the proposed SIRT-FBP-MS-D-DIFF algorithm. During the data reconstruction, the dynamic scans are automatically aligned to the static scan using cross-correlation. After the alignment, the static sinogram is subtracted from the dynamic sinograms to obtain difference sinograms containing only dynamic changes and noise^17^. For fully dynamic samples (for example foams), the sinogram subtraction step can be omitted as the measured sinograms contain only dynamic changes. The difference sinograms are automatically reconstructed by the SIRT-FBP algorithm (“SIRT-FBP” section in “Background” section) to reconstruct the extracted dynamic features. This allows to obtain the reconstruction quality of SIRT with the computation time of a standard FBP. Moreover, as the SIRT filter is combined with an analytical reconstruction algorithm, interior tomography datasets can be simply accommodated by standard edge-padding^34^ without need for a more complex and time-consuming virtual sinogram step typically required if iterative reconstruction techniques are used^17^. For a more detailed description of the sinogram alignment and subtraction steps we refer to^17^. The reconstruction step is directly followed by application of a trained MS-D network (“Mixed-scale dense convolutional neural network” section in “Background” section) to extract the dynamic features from the reconstructed data volumes in an automatic and efficient manner.

#### Network training

**Figure 1:**
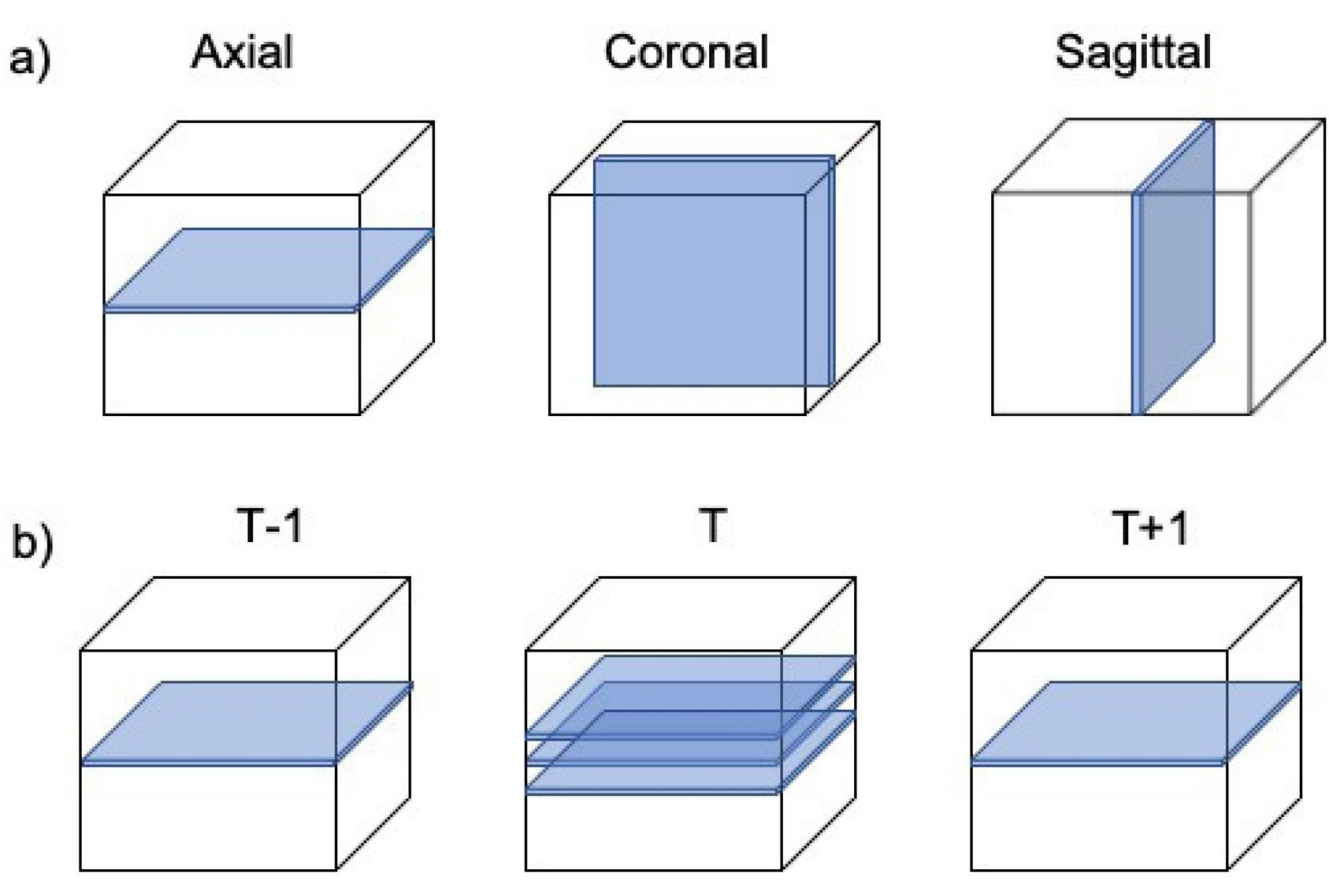
Schematic illustration of the training schemes. (**a**) Axial, coronal and sagittal views of the sample. The axial view is used during standard training. For the ensemble training, all three views are individually trained. (**b**) The quasi-4D training scheme. For each time step T, the adjacent slices in time- and volumetric direction are considered.

To establish the optimal network training scheme for water classification, three different training protocols for the MS-D network were considered: standard network training, quasi-4D network training taking into account time (1D) and volume (3D) simultaneously, and an ensemble of three independently trained networks. For each training protocol (Fig. 1), the SIRT-FBP reconstructions of the difference sinograms were considered as the network input. The target images were created by using the iterative reconstruction pipeline proposed by Bührer et al.^17^, designed specifically to extract dynamic features from a static matrix by combining iterative reconstruction and time-regularization.

A *standard MS-D network* was trained by pairing the single input and target slices ${{\varvec{x}}}_{i}^{\mathrm{input}}$ and ${{\varvec{x}}}_{i}^{\mathrm{target}}$, where $i \in 1,\dots ,N/2$ corresponds to the horizontal slices of the volumes ${{\varvec{x}}}^{\mathrm{input}}$ and ${{\varvec{x}}}^{\mathrm{target}}$ (Fig. 1a, axial). The redundant information of the time- and/or volume-sequence is not exploited during training.

A *quasi-4D network* was trained by collecting the input slices into slabs and training the network between the input slabs and single target slices. The slabs were created by considering for each input image ${{\varvec{x}}}_{i,t}^{\mathrm{input}}$ at slice $i$ and time step $t,$ the adjacent slices in the time and volume dimension: ${\left[{\left[{{\varvec{x}}}_{j,p}^{\mathrm{input}}\right]}_{j=i-1}^{i+1}\right]}_{p=t-1}^{t+1}$ where $t\in 1,\dots ,T$ corresponds to the acquired time frames and $i$ to the number of slices per volume (Fig. 1b). This enabled the network to simultaneously exploit the time and volumetric information available in the acquired dynamic time-series of volumetric data. To ensure the same slab size for all input slices, symmetric padding was used at the time and volume borders. It is important to note that the network can, in principle, be provided with slabs of any size, though the training time and required memory increase with slab size. In this work the slabs were limited to the single-adjacent slices in the volume- and time-sequences, leading to a slab size of 5 (Fig. 1b) due to memory constraints.

An *ensemble network* training (majority voting) was considered as a final training scheme. For this, three different standard MS-D networks were trained independently by selecting three different views of the data volume (Fig. 1a) as proposed in^41^. After training, the networks were independently applied to the datasets, and the corresponding network outputs were merged. During merging, the pixels labeled as dynamic features by all trained networks were labeled as dynamic while the other pixels were set to zero to obtain the final segmentation by the ensemble network.

Following the MS-D network model presented in^19^, a network consisting of 100 intermediate layers was initialized for each training scheme and the convolution (3 × 3 pixel kernel) at each layer $i$ was dilated by ${d}_{i}=1+(i \,\mathrm{mod} 10)$. The number of channels per layer was always 1 and the number of input channels for the quasi-4D approach was 5 (Fig. 1). A rectified linear unit (ReLU) activation function has been used in the intermediate layers, while for the final output layer a softmax activation has been chosen. For a more detailed description of the underlying network model, including a schematic figure, we refer to^19^.

The network was trained for 100 epochs using the ADAM algorithm with mini-batch size of 1 (single image), which minimizes the negative log-likelihood training loss between the network output and input images after each epoch. On a separate validation dataset, the validation error was evaluated through the negative log-likelihood after each epoch and the training epoch yielding the lowest validation error was chosen as the optimal trained network.

#### Algorithm protocol

**Figure 2:**
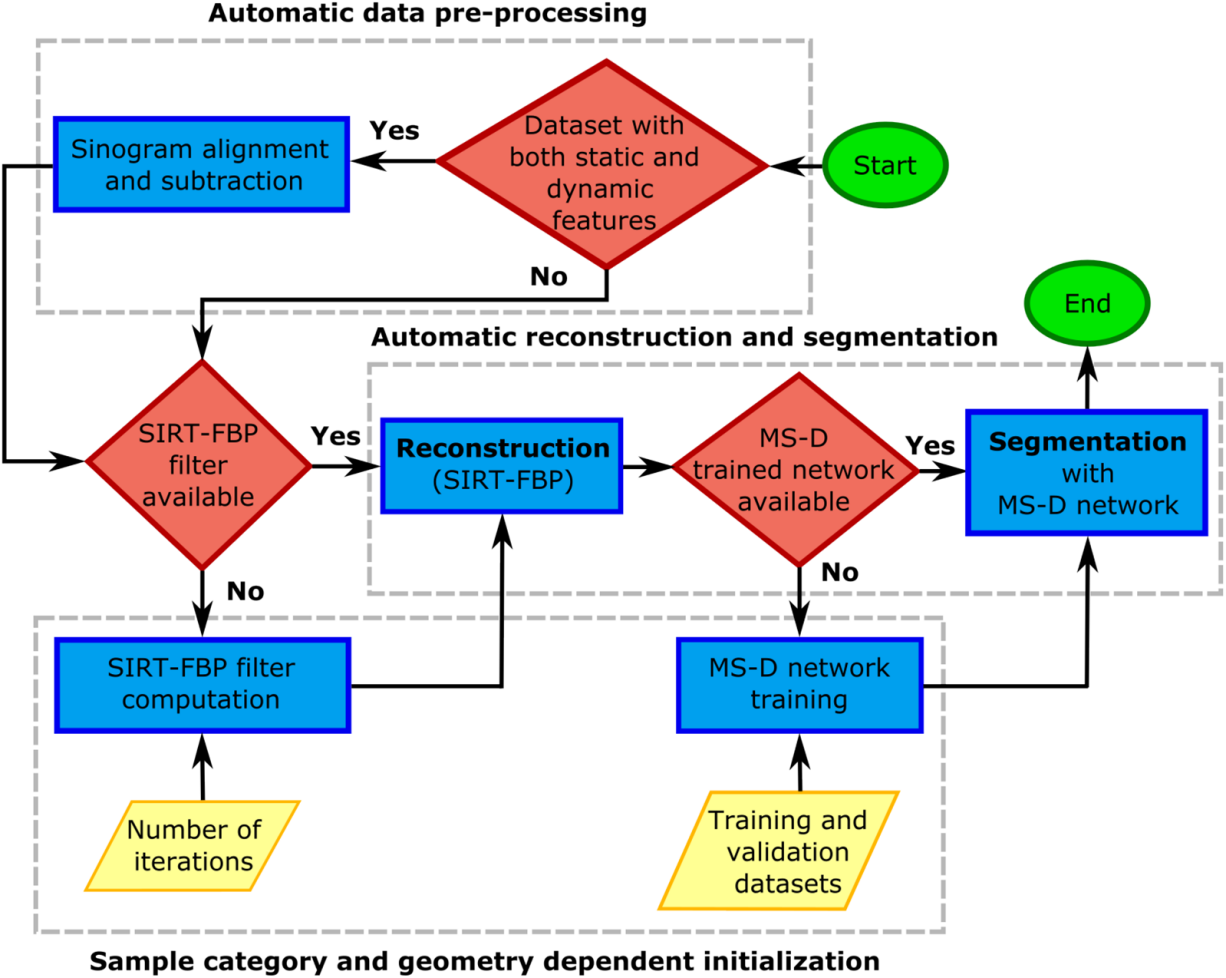
Algorithm protocol flowchart. The algorithm protocol starts with an automatic data pre-processing step to extract the dynamic components. For each experiment geometry a SIRT-FBP filter needs to be computed, and for each sample category (e.g. fuel cell, porous media, foams) a MS-D network trained. If these 2 elements are available, reconstruction and feature extraction can be performed fully automatically.

To apply the proposed SIRT-FBP-MS-D-DIFF algorithm, we propose the following protocol presented in Fig. 2:

0. Pre-requisite:
  - Set the number of SIRT iterations that will be approximated by the filter.
  - Prepare a representative dataset for network training consisting of raw SIRT-FBP reconstructions of difference sinograms (steps 1.a, 2, 3.a below) (input) and the corresponding segmentations (target). The imaging conditions and sample type (e.g. a fuel cell, a catalyst) should represent the datasets to which the trained network will be applied to.
1. Pre-processing:
  - Geometry dependent: pre-compute the angle-dependent SIRT-FBP filters (“SIRT-FBP” section in “Background” section).
  - Geometry independent: train the MS-D network with a training dataset (“Network training” section in “SIRT-FBP-MS-D-DIFF” section).
2. Align and subtract sinograms to extract the dynamic changes (“Data reconstruction” section in “SIRT-FBP-MS-D-DIFF” section).
3. Reconstruct and extract the dynamic features with the SIRT-FBP-MS-D-DIFF algorithm:
  - Run SIRT-FBP on the difference sinograms to reconstruct the dynamic features and noise.
  - Apply the trained MS-D network to automatically classify dynamic features.

The network has to be trained with an initial training dataset consisting of raw reconstructions (input) and the corresponding segmentations (target). The segmentations used for network training can be created depending on user preference, by using the previously proposed rSIRT-PWC-DIFF algorithm protocol^17^ or any other segmentation pipeline. In addition, it is necessary to select the number of SIRT iterations approximated by the filter (see step 0.a above). For this, following the automatic stopping criterion proposed by Bührer et al.^17^ a set of SIRT-FBP reconstructions can be computed for a selection of iterations (for example 10, 20, …, 700). Between the adjacent increase in iteration, the difference between the corresponding reconstructions can be measured through the Euclidean L2-norm. By normalizing the resulting L2-norms and measuring their gradient, the gradient slope position can be chosen as the optimal number of iterations to approximate in SIRT-FBP. Though this requires computation of several SIRT filters, once the iteration number is chosen, the corresponding pre-computed filter can be directly applied to all datasets with computational efficiency of a standard FBP. For more a detailed explanation of the stopping criterion we refer to^17^.

## Materials

The algorithm performance was assessed on three dynamic Polymer Electrolyte Fuel Cell (PEFC) synchrotron tomographic datasets (Supplementary information Fig. S1 online) collected from two different cells at the TOMCAT beamline at the Swiss Light Source (SLS) at the Paul Scherrer Institut, Switzerland^42^. Both cells had a diameter of approximately 5 mm. The setup for all experiments consisted of a high-numerical-aperture macroscope^43^ (fixed 4× magnification), a 150 μm thick LuAG:Ce scintillator (Crytur, Turnov, Czech Republic) to convert the X-rays into visible light and the in-house developed high-frame rate GigaFRoST detector^44^. The experiments were performed with filtered (20 mm Sigradur and 75 μm Mo) polychromatic radiation with a peak energy of approximately 20 keV. To reach the required time resolution, the horizontal field of view was cropped to approximately 4 mm, leading to interior tomography datasets. The collected images were 1440 × 1100 pixels with a pixel size of 2.75 μm.

During data acquisition, both cells were connected from above with two long tubes to simultaneously ensure a continuous gas flow to the cell and continuous cell rotation. 300 evenly distributed projections of the cells were collected between 0° and 180° with no image blurring despite continuous sample rotation during data acquisition. For all datasets the exposure time was set to 0.3 ms for each projection image, resulting in a total scan time of 0.1 s per tomogram.

The first dataset (PEFC_1) was collected from a cell (flow field plates made of BMA5, SGL Technologies; membrane electrode assembly: Gore Primea A510.1/M815.15/C510.4 with a 15 µm thick membrane and anode/cathode Pt loadings of 0.1/0.4 mg/cm^2^; GDL: Toray TGP-H-060, Toray Industries Inc.) by acquiring a total of five sets of tomographic datasets, each consisting of 60 continuous time steps. The data for the first 3 sets (3 × 60 scans) are available in TomoBank^45^. A set of 30 continuous time steps (time steps 121 to 150) with water in the gas diffusion layer was then chosen for the network training. A single dry scan with the same experimental parameters was considered for the sinogram alignment and subtraction. The total experiment time (time steps 1–300) was 4 min.

The second dataset (PEFC_2)^46^ was collected using the same cell as in the PEFC_1 dataset. A total of 49 time frames were collected. Unlike PEFC_1, the time series was not continuous, but for the time steps 1–41 the scans were collected every 3 s and for the time steps 42–49 every 30 s. At the beginning of the time series the cell was completely dry, having water appearing within the cell during the time sequence. The first time step was therefore considered as a dry scan for the sinogram alignment and subtraction step. The total experiment time was 7 min.

The third dataset (PEFC_3)^46^ was collected from a different cell (materials identical to PEFC_1 except for the GDL: Freudenberg I6, Freudenberg Group, Germany) with smaller GDL average pore size. The acquisition strategy was identical to the PEFC_2 dataset: a total of 49 time steps were collected with 3 s time gap between time steps 1–41 and 30 s gap for time steps 42–49, leading to a total experiment time of 7 min. As for PEFC_2, the cell was dry at the beginning of the time series. Therefore, the first time step was considered as a dry scan during sinogram alignment and subtraction.

All projection images were dark and flat-field corrected. Phase retrieval of each projection with the Paganin algorithm^47^ followed before reconstruction. The phase retrieval algorithm, implemented in the reconstruction pipeline available at TOMCAT^48^, was applied together with a deconvolution step to enhance the contrast between different materials while minimizing compromise in the spatial resolution^49^.

## Results

### Training and validation dataset

Each network was trained on the PEFC_1 dataset (“Materials” section) cropped to the region of interest (dynamically active area). Each PEFC_1 time step volume was split from the middle into training and validation sets, so obtaining 30 training and 30 validation volumes each of size 237 × 1407 × 236 pixels. The target segmented images for training were obtained by applying the iterative reconstruction pipeline described in^17^.

When applying the trained networks, the confidence threshold was set to 20%, causing voxels to be classified as dynamic if their assigned probability of being dynamic exceeds 20%. This ensures a more balanced true and false positive rate, which is necessary because of the substantial class imbalance in the dataset (only 5% of the voxels are dynamic)^50^.

The lowest validation error for all networks was actually reached already after 25 (“Standard”) to 55 (“Quasi-4D”) epochs since the used data is rather simple (only dynamic signal and background noise). After this minimum, the error kept oscillating within the neighborhood of its lowest value until the maximum 100 epochs were reached. This indicates that the overfitting risk is minimal in practice and that the training time can be reduced by stopping earlier using the validation error as guidance.

### Metrics and evaluation

Each training scheme was evaluated by applying the trained network, without re-training, to two test sets (PEFC_2 and PEFC_3, “Materials” section) and to the training set (PEFC_1). The resulting segmented volumes were compared to independent manual segmentations obtained from a manual ad-hoc post-processing pipeline^13^. The network segmentations were evaluated through sensitivity, specificity^51^, false-positive-rate, false-negative-rate^52^ and Dice coefficient^53^.

### Software

The algorithm was developed in Python 3.7. The SIRT-FBP^34^ algorithm was used together with the ASTRA toolbox^54–56^ to implement the reconstruction step and the PyTorch^57^ implementation of the MS-D network^23^ was used for the segmentation step. A single Nvidia Tesla V100 GPU card was exploited to accelerate computations.

### Results

**Figure 3:**
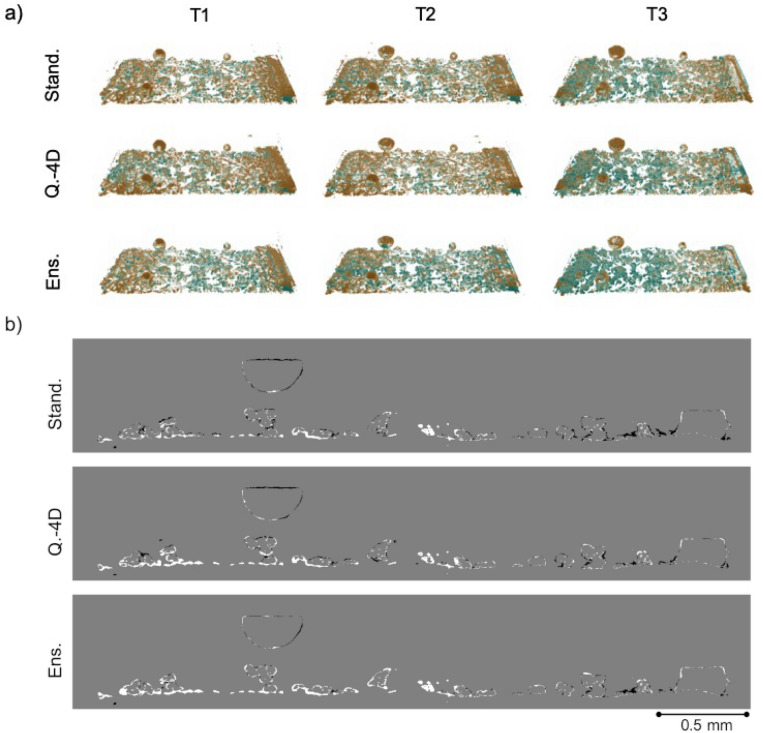
Comparison of the recovered water for the PEFC_1 dataset. The first column presents the first time step (T1), the second column time step 15 (T2) and the third column time step 30 (T3). (**a**) Difference volumes between the manual segmentation (ground truth) and SIRT-FBP-MS-D-DIFF water classifications with standard (Stand.), quasi-4D (Q.-4D) and ensemble (Ens.) training schemes of the MS-D network, respectively. The green color corresponds to features present only in the ground truth, orange to features present only in the SIRT-FBP-MS-D-DIFF water classifications. (**b**) Difference slice (slice 143, T3) between the water classification with the networks and ground truth segmentation. The white structures correspond to features present only in the ground truth segmentation, black structures only in the SIRT-FBP-MS-D-DIFF water classification.

**Figure 4:**
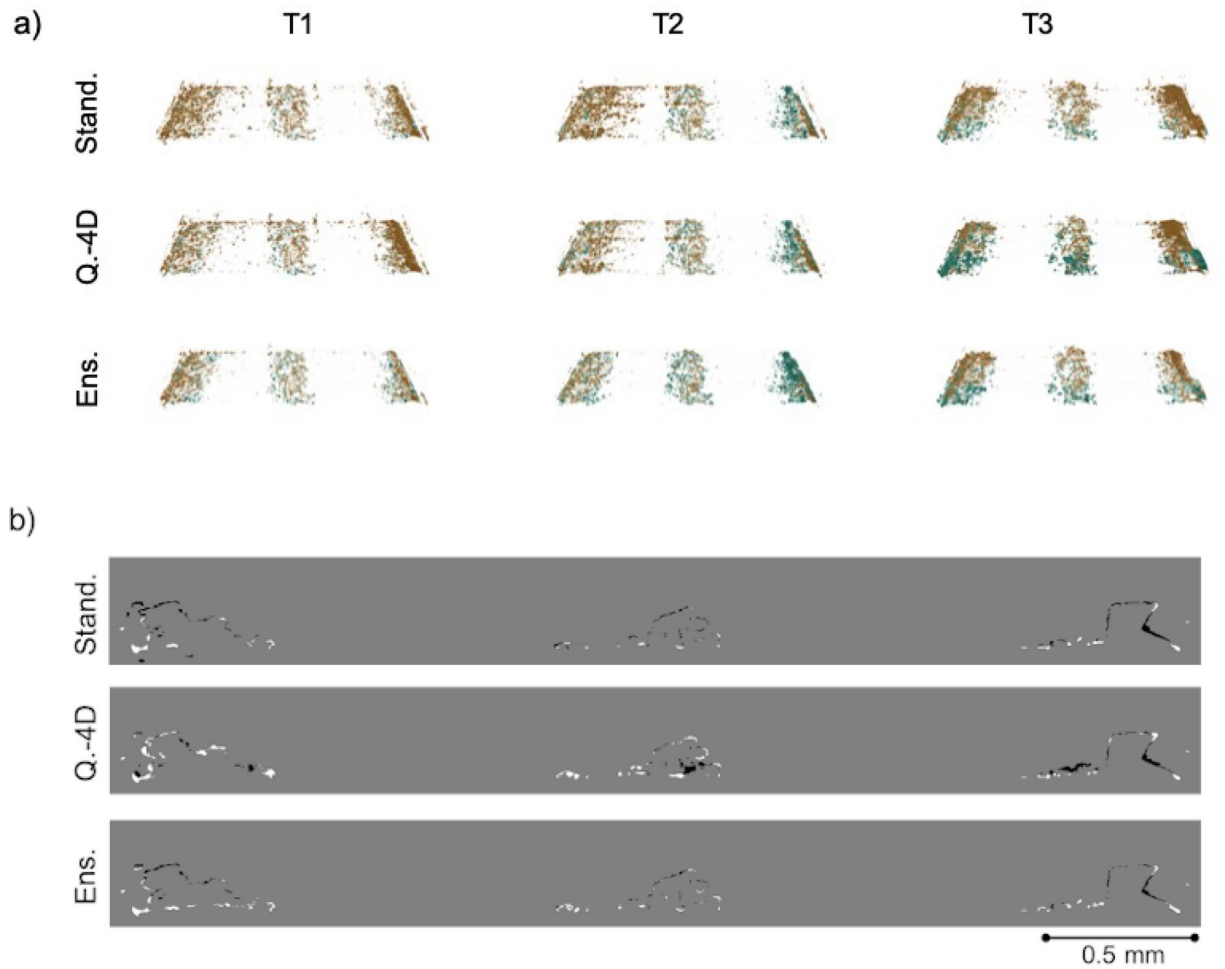
Comparison of the recovered water for the PEFC_2 dataset. The first column presents the time step 25 (T1), the second column time step 37 (T2) and the third column time step 49 (T3). (**a**) Difference volumes between the manual segmentation (ground truth) and SIRT-FBP-MS-D-DIFF water classifications with standard (Stand.), quasi-4D (Q.-4D) and ensemble (Ens.) training schemes of the MS-D network, respectively. The green color corresponds to features present only in the ground truth, orange to features present only in the SIRT-FBP-MS-D-DIFF water classifications. (**b**) Difference slice (slice 563, T3) between the water classification with the networks and ground truth segmentation. The white structures correspond to features present only in the ground truth segmentation, black structures only in the SIRT-FBP-MS-D-DIFF water classification.

**Figure 5:**
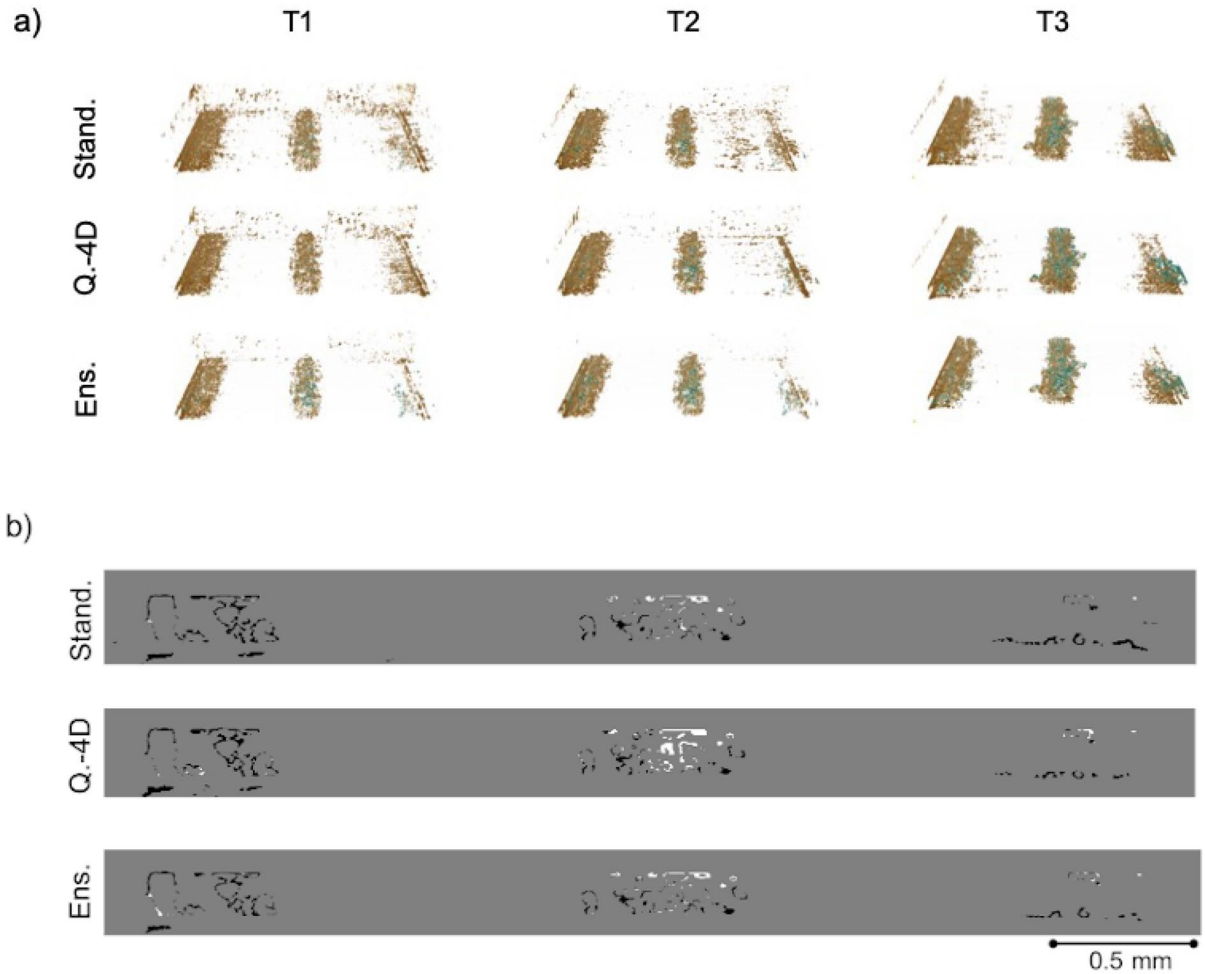
Comparison of the recovered water for the PEFC_3 dataset. The first column presents the time step 25 (T1), the second column time step 37 (T2) and the third column time step 49 (T3). (**a**) Difference volumes between the manual segmentation (ground truth) and SIRT-FBP-MS-D-DIFF water classifications with standard (Stand.), quasi-4D (Q.-4D) and ensemble (Ens.) training schemes of the MS-D network, respectively. The green color corresponds to features present only in the ground truth, orange to features present only in the SIRT-FBP-MS-D-DIFF water classifications. (**b**) Difference slice (slice 553, T3) between the water classification with the networks and ground truth segmentation. The white structures correspond to features present only in the ground truth segmentation, black structures only in the SIRT-FBP-MS-D-DIFF water classification.

The SIRT-FBP-MS-D-DIFF classified water volumes of the three cells PEFC_1, PEFC_2 and PEFC_3 and the corresponding manual segmentations are presented in Figs. 3a, 4a and 5a, respectively. The classified water volumes are presented for all three network training protocols: standard, quasi-4D and ensemble. The manual segmentations are used as ground truth to evaluate the accuracy of each training scheme. The difference volumes between the classifications and the ground truth are presented in Figs. 3b, 4b and 5b, respectively. For all samples, the manual segmentations were generated by aligning the dynamic Gridrec reconstructions to an additional high-quality Gridrec reconstruction of the cell in a dry state (without dynamics) (1000 projections with 1 ms exposure time) of the sample followed by image subtraction to isolate the dynamic features (water) from the static matrix^16^. The subtracted images were further post-processed to obtain the final segmentations for each time frame^13^. In addition, static masks were created by segmenting the GDL fiber structures and applied to the manually segmented volumes within the post-processing pipeline to exclude any fiber structures misclassified as water^13^. To allow comparison to the manual segmentations, the same masks were also applied to all SIRT-FBP-MS-D-DIFF reconstructions. The reconstructions were evaluated through sensitivity, specificity, false-positive-rate, false-negative-rate and dice metrics, results are presented in Table 1.

**Table 1:** Comparison of the classified water accuracy for the volumes at time steps T1, T2 and T3 computed between the SIRT-FBP-MD-D-DIFF and a manual segmentation (ground truth) for each MS-D network design and fuel cell dataset (PEFC_1 training and validation, PEFC_2, PEFC_3).

Network	Dataset	Dice			Sensitivity		
		T1 (%)	T2 (%)	T3 (%)	T1 (%)	T2 (%)	T3 (%)
Standard	PEFC_1, training	87	88	88	92	93	88
	PEFC_1, validation	88	87	86	94	91	86
	PEFC_2	79	82	88	95	91	95
	PEFC_3	55	70	80	97	96	97
Quasi-4D	PEFC_1, training	86	89	87	91	94	86
	PEFC_1, validation	87	89	84	92	93	80
	PEFC_2	78	84	84	94	88	86
	PEFC_3	58	71	80	98	96	93
Ensemble	PEFC_1, training	88	88	87	90	88	83
	PEFC_1, validation	89	86	84	91	83	77
	PEFC_2	84	85	90	90	82	92
	PEFC_3	69	77	85	93	94	94

The classified water accuracy was measured through dice coefficient and sensitivity throughout the volume.

As can be seen from Fig. 3a, the difference volumes between the water classifications and ground truth segmentation demonstrate strong similarity between the three training methods’ performance. For the ensemble training scheme some reduction in falsely reconstructed water (orange features) can be seen at the top surfaces of the water features across all time steps. The differences in the dice metric between all methods (Table 1) are limited to maximum 2% points, so supporting the high correlation between all methods’ performance. The sensitivity was found to be maximized by the quasi-4D method for time step T2 and by the standard method for T3. Specificity was found to be close to 100% for all time steps and methods, leading consecutively to false-positive-rates of nearly 0%. For all training schemes 1% increase in sensitivity and dice were detected at T1 for the validation half of the volume. This is due to a slight registration inaccuracy in favor of the validation dataset. The location of the falsely reconstructed water features (Fig. 3a orange, 3b black) and missing water features (Fig. 3a green, 3b white) were identified at the top and bottom boundaries of the water features. Figure 3b further reveals that the size and boundaries of the water features are in slight disagreement between the SIRT-FBP-MS-D-DIFF classifications and the manual segmentations. The accuracy metrics in Table 1 can therefore be considered as a slightly pessimistic evaluation of the water detection accuracy due to the combined effect of the size and boundary difference. The classified water volumes of the ground truth and the different training schemes are presented in Supplementary information Fig. S2 online.

For comparison, the same accuracy metrics were also computed between the SIRT-FBP-MS-D-DIFF water classifications and the target volumes used to train and validate the networks. The target volumes were computed with the rSIRT-PWC-DIFF algorithm^17^. The accuracy metrics were evaluated separately for the training and validation halves of the PEFC_1 water classifications, results presented in Table 2. The dice metric is in high agreement for all three training schemes at all time steps, quasi-4D reaching the highest performance at T2 and ensemble method only slightly improving the quasi-4D and standard methods at time step T3. The sensitivity is highest at time steps T1 and T2 when the quasi-4D method is used, increasing from the standard method on average by 2%. At time step T3, the standard method yields the best performance, reaching at maximum 9% points higher performance on the validation half than the ensemble method. The specificity was found to be between 98 and 99% for all training schemes and time steps, leading consecutively to false-negative rates of 1–2% across the time sequence. Though the target and reconstructed volumes were now automatically aligned and additional registration was not needed, similar slight disagreement of the water feature sizes as detected between the classification results and manual segmentations (Fig. 3b) was observed.

**Table 2:** Comparison of the classified water accuracy for the volumes at time steps T1, T2 and T3 computed between the SIRT-FBP-MD-D-DIFF and target volumes used for network training and validation.

Network	Dataset	Dice			Sensitivity		
		T1 (%)	T2 (%)	T3 (%)	T1 (%)	T2 (%)	T3 (%)
Standard	PEFC_1, training	80	87	85	91	92	94
	PEFC_1, validation	76	82	80	93	91	92
Quasi-4D	PEFC_1, training	83	89	86	93	95	92
	PEFC_1, validation	77	84	81	94	93	88
Ensemble	PEFC_1, training	82	88	87	88	87	89
	PEFC_1, validation	79	82	81	89	83	83

The target volumes were computed with the rSIRT-PWC-DIFF algorithm^17^. The results are shown separately for each MS-D network design, further split between the training and validation halves of the PEFC_1 fuel cell dataset. The classified water accuracy was measured through dice coefficient and sensitivity through the volume.

For the test dataset PEFC_2, the performance in Fig. 4 is similar for the different training schemes, while the ensemble method is able to successfully suppress speckled noise (Fig. 4a) at all time steps (for classified water volumes see Supplementary information Fig. S3 online). The metrics in Table 1 reveal that the dice coefficient is highest across the time sequence for the ensemble method, reaching maximum 6% points increased performance at time steps T1 and T3 than the quasi-4D method. The highest sensitivity was reached by the standard method for all time steps, with maximum difference of 9% points at time step T2 to the ensemble method. Qualitative comparison for PEFC_3 agrees with the qualitative evaluation of PEFC_2: Fig. 5 reveals that while the methods reach similar segmentations, the speckled noise is significantly suppressed with the ensemble training scheme (for classified water volumes see Supplementary information Fig. S4 online). This is supported by the dice metric (Table 1) which reaches the highest values across the time sequence by the ensemble method. As with the PEFC_2 dataset, the sensitivity metric, though similar for all methods, is slightly higher with the standard method, increasing from the ensemble method on average by 3% points and the quasi-4D method by 1% point.

For both test datasets similar water droplet boundary effects as with PEFC_1 can be observed in Figs. 4b and 5b: the differences between the ground truth and water classifications are strongly focused on the water feature boundaries, typically the upper boundaries tending to overestimate the water while the lower boundaries are suppressed. This is further demonstrated in Table 3, where the maximum water voxels for the segmentation and SIRT-FBP-MS-D-DIFF water classifications are presented. When the exact position of the voxels is discarded and only the absolute number of water voxels is considered, the percentage of the recovered water increases significantly for most time steps. While all networks have tendency to over-estimate the water distribution for PEFC_3, the ensemble training scheme is able to reach a water content closest to 100% by suppressing uncertainties caused by noise (speckled structures in Fig. 4a). For the training dataset PEFC_1, the standard method is able to reach a water content closest to 100% at time step T3.

**Table 3:** Water volume comparison between the volumes at time steps T1, T2 and T3 computed between the SIRT-FBP-MD-D-DIFF classifications and a manual segmentation (ground truth) for each MS-D network design and fuel cell dataset (PEFC_1 training and validation, PEFC_2, PEFC_3).

Network	Dataset	Maximum recovered water volume		
		T1 (%)	T2 (%)	T3 (%)
Standard	PEFC_1, training	111	112	97
	PEFC_1, validation	113	108	99
	PEFC_2	140	120	117
	PEFC_3	250	177	141
Quasi-4D	PEFC_1, training	110	106	95
	PEFC_1, validation	112	109	91
	PEFC_2	139	111	106
	PEFC_3	239	171	131
Ensemble	PEFC_1, training	105	100	90
	PEFC_1, validation	104	94	84
	PEFC_2	113	94	105
	PEFC_3	170	142	121

The maximum classified water volume was calculated as a number of water voxels present in the SIRT-FBP-MD-D-DIFF classified volumes compared to the number of ground truth water voxels.

The strong similarity between the water classifications obtained from the three training approaches reveals that the different training schemes converge to a similar final water extraction profile. This similar convergence is suspected to arise from the relative simplicity of the training problem: as the networks are trained on the difference volumes, the training volumes contain only dynamic features (here water), noise and possible reconstruction artefacts. As no complex static features are present, it is relatively easy for each network to learn extracting the single dynamic phase from the background. Due to the simplicity of the training volumes and their relative sparsity, the additional information leveraged by the quasi-4D and ensemble methods remains minor, leading to a highly similar performance for the PEFC_1 (Fig. 3) compared to the standard approach. As the ensemble method was able to reach higher similarity between the classified water and ground truth, demonstrated both by the dice metrics (Table 1) and the total recovered water volume (Table 2) for all three datasets and visually suppress noise artefacts especially for the PEFC_2 and PEFC_3 datasets (Figs. 4, 5), we recommend to use the ensemble network scheme to optimize the obtainable reconstruction accuracy. When aiming to perform a quick initial quality check of the obtained data to, for instance, prioritize data processing or evaluate data quality, we recommend the standard network scheme to minimize the data processing time while the classification accuracy is only slightly compromised.

## Discussion

We have introduced the SIRT-FBP-MS-D-DIFF algorithm designed to efficiently reconstruct and segment dynamic, low SNR interior tomography data volumes containing dynamic features potentially without prior knowledge of the sample composition and its inner features. The proposed algorithm is inspired by the reconstruction protocol described in^17^. The dynamic features are extracted prior to reconstruction through difference sinograms between the static and dynamic tomograms to directly extract dynamic features in an automated manner. The difference sinograms are efficiently reconstructed through the SIRT-FBP algorithm^34^ that successfully approximates SIRT reconstructions with an angle-dependent filter within a standard analytical reconstruction algorithm, reaching comparable computation time as a standard FBP reconstruction. The raw reconstructions are directly post-processed through a trained neural network to efficiently extract the dynamic features from noise and possible reconstruction artefacts. Prior knowledge and input required by the algorithm protocol have been strongly minimized while striving for high computational efficiency to allow the algorithm application to large data volumes with hundreds of time steps and to data of different samples of similar type (for example other fuel cells). Though the algorithm protocol has been demonstrated on fuel cell datasets consisting of static and dynamic phases, we expect that the same protocol can be applied to any datasets with one single evolving phase including fully dynamic samples (e.g. foam) by training the network with a representative dataset. After training, the algorithm can be directly applied to other similar types of datasets without additional retraining or parameter tweaking.

Even though the algorithm has been tested only with different fuel cell datasets, we envisage that for systems with a static and dynamic part, where difference sinograms are required, the resulting subtracted datasets with only the dynamic fraction (e.g. water) can have similar characteristics even for very different systems (e.g. full cells and water evolution in sandstone), for which the same trained model could potentially produce satisfactory results. More extensive training with a variety of datasets for different samples could potentially also help in improving the reliability of the network. This point needs though to be tested and verified, but is beyond the scope of this work.

To optimize the computation time, it is recommended to pre-compute the necessary angle-dependent SIRT filters for the reconstruction. Once the filters are computed, the reconstruction pipeline can be applied in parallel to all measured data in the computation time of FBP. Alternatively, the sinograms can be aligned into the same angular range for all data volumes to enable the use of a single computed filter, corresponding to this specific angular range, to all measured data.

As demonstrated with the three fuel cell datasets, the MS-D network was trained using only a single dataset (PEFC_1). Though the training dataset had significantly larger dynamic content compared to the other datasets, we demonstrated direct applicability of the algorithm to the testing datasets (PEFC_2 and PEFC_3, Figs. 4, 5) without network retraining. The trained networks were able to classify water with 80–97% sensitivity (Table 1) and reach similar water volume content (Table 3) as the manual segmentations which were used to estimate the ground truth water content. Over 90% water sensitivity was reached even for the PEFC_3 dataset, for which the cell materials consisted of significantly smaller GDL structures and pores, leading to a completely different water distribution profile in 3D (“Materials” section). In the future, the performance on PEFC_3 could even be further optimized by including smaller water volume content within the training dataset so to maximize its representativeness. These results demonstrate how, by exploiting difference sinograms to simplify the training problem, it is possible to train the network just once on a single target dataset and apply it directly to other similar datasets without network re-training, so drastically reducing the required training time.

In time-resolved X-ray tomographic microscopy investigations, such as fuel cell imaging experiments, the collected datasets typically represent complicated sample structures, but as the SNR of the collected tomograms is highly limited, their reconstruction and post-processing to extract dynamic components is often very challenging. The post-processing pipelines often require several processing steps such as data reconstruction, registration, filtering and segmentation, each step requiring typically manual parameter tuning and optimization. Unfortunately, the manually tuned parameters do not generalize well, thus requiring new manual parameter evaluation when the experimental conditions or the imaged samples are modified. This manual work requirement extends the total data post-processing time significantly, posing limits to the feasible number of samples and time steps that can be realistically post-processed. The proposed algorithm protocol has been designed to obtain a significant reduction in manual labor while obtaining accurate feature extraction rates on limited computation resources.

When 100 full volume time steps of good quality data of a single fuel cell are processed with the current pipeline including manual parameter tuning and optimization, the complete processing from raw data to segmented water requires in the best case 2 weeks of work (using 15 CPU nodes). The work is though significantly dominated by the unavoidable manual labor, not by the computation. If the image quality is challenging or a completely new cell type and material are being investigated, additional manual tuning and parameter search might be necessary, causing the processing time to extend from 2 weeks up to 1 month. This processing time was successfully decreased by the rSIRT-PWC-DIFF algorithm^17^ which was able to process a similar data quantity fully automatically in 1 week. The algorithm protocol proposed here can further reduce the processing time to approximately 7.2 h on a single GPU node when the standard network scheme is used. This processing time is divided to 5.8 h for the sinogram alignment, 20 min for the reconstruction step and 1.1 h for the feature extraction through a single MS-D network. With the ensemble MS-D network, considering a single GPU card, approximately 1.5 h additional time for the feature extraction is necessary to apply the three trained networks. Additional computation time is required for network training and the angle-dependent SIRT filter calculation. On the single GPU node used, the standard network training for 100 epochs requires approximately 29 h, coronal view 26 h, sagittal view 110 h and quasi-4D 106 h when training on 30 time step volumes, each consisting of 600 slices. To characterize the algorithm performance, the training was completed for 100 epochs in all cases. As with the current training data, the lowest validation error was reached already before half of the total number of epochs, in the future the training time could be further optimized by developing an automatic stopping criterion. Since the network training and filter calculation are completely independent, they can be performed in parallel on multiple GPU cards to further reduce the total additional computation time. Once the angle-dependent filters are calculated and the network is trained, the same trained network and filters can be used for other datasets without re-computation, reducing the processing time back to the aforementioned 7.2 h. Thanks to the high computational efficiency, the proposed protocol can be applied on limited computational resources in an automatic manner to significantly accelerate the required data processing time. This is foreseen to open up new possibilities in dynamic X-ray tomographic investigations as a considerably larger parameter space can be efficiently handled. Moreover, the full automation of the proposed algorithm and parallelized code create ideal conditions to distribute the computations to a supercomputing facility, significantly expanding the possible future data processing applications.

## Supplementary Information

Supplementary Information.

## Data Availability

The PEFC_1 dataset is available in TomoBank (https://tomobank.readthedocs.io/en/latest/source/data/docs.data.dynamic.html#fuel-cell-data). The PEFC_2 and PEFC_3 datasets are available upon request. The PyTorch implementation of the standard MS-D network is available in Github (https://github.com/ahendriksen/msd_pytorch). The standard SIRT-FBP implementation is available in Gitlab (http://dmpelt.gitlab.io/sirtfilter/). The SIRT-FBP-MS-D-DIFF implementation is available upon request.
