# Supplementary material for: Deep learning based classification of dynamic processes in time-resolved X-ray tomographic microscopy
Source: Sci Rep. 2021 Dec 17;11:24174. doi: 10.1038/s41598-021-03546-8 (PMC8683503; doi:10.1038/s41598-021-03546-8)
Supplement: Supplementary file 1 — Supplementary Information. [file 41598_2021_3546_MOESM1_ESM.docx]

Supplementary information: Deep Learning Based Classification of Dynamic Processes in Time-Resolved X-ray Tomographic Microscopy

*Minna Bührer^a,b^, Hong Xu^c^, Allard A. Hendriksen^d^, Felix N. Büchi^c^, Jens Eller^c^, Marco Stampanoni^a,b^, Federica Marone^a*^*

*^a^Swiss Light Source, Paul Scherrer Institut*

*^b﻿^Institute for Biomedical Engineering, University and ETH Zürich*

*^c^Electrochemistry Laboratory, Paul Scherrer Institut*

*^d^Centrum Wiskunde & Informatica, Amsterdam, The Netherlands.*

**federica.marone@psi.ch*

S1. Materials


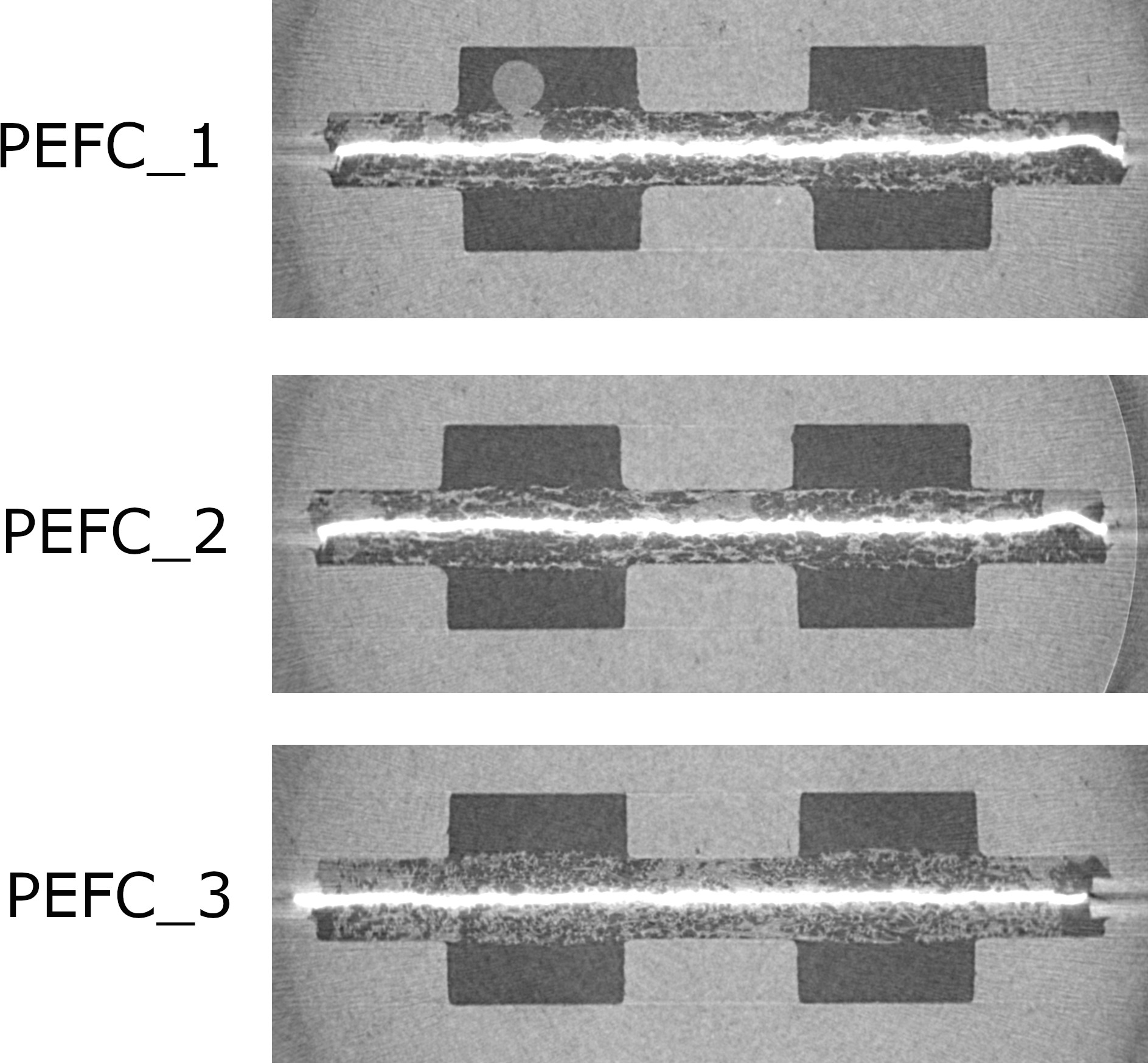


Figure S1. Phase retrieved ^54^ gridrec reconstructed slices of the three fuel cell datasets cropped to the region-of-interest. The datasets PEFC_1 and PEFC_2 show the same cell with different water distributions. The PEFC_3 is a different cell with a smaller fiber structure in its gas diffusion layer (GDL). The PEFC_1 dataset was used for training and validating the trained networks. The PEFC_2 and PEFC_3 datasets were used to test the reconstruction pipeline without network retraining.

S2. Reconstruction results

The following figures present the water classification results for the fuel cells PEFC_1, PEFC_2 and PEFC_3.


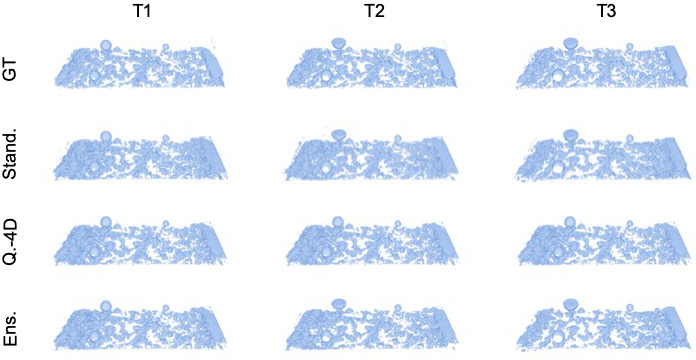


Figure S2. Comparison of the recovered water for the PEFC_1 dataset. The first column presents the first time step (T1), the second column time step 15 (T2) and the third column time step 30 (T3). The rows correspond to the ground truth manual segmentations and SIRT-FBP-MS-D-DIFF water classifications with standard, quasi-4D and ensemble training schemes of the MS-D network, respectively.


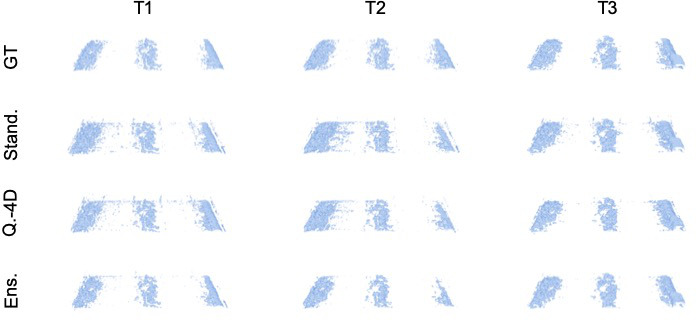


Figure S3. Comparison of the recovered water for the PEFC_2 dataset. The first column presents the time step 25 (T1), the second column time step 37 (T2) and the third column time step 49 (T3). The rows correspond to the ground truth manual segmentations and SIRT-FBP-MS-D-DIFF water classifications with standard, quasi-4D and ensemble training schemes of the MS-D network, respectively.


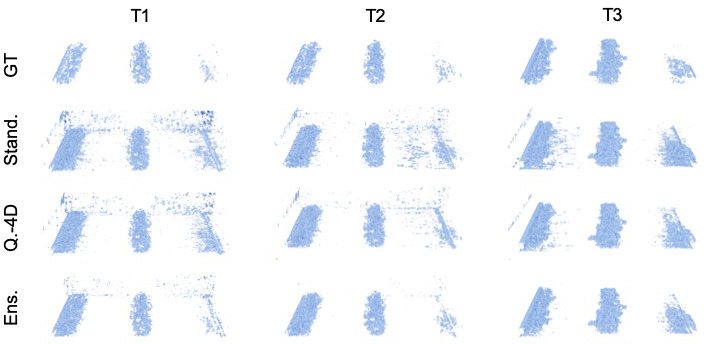


Figure S4. Comparison of the recovered water for the PEFC_3 dataset. The first column presents the time step 25 (T1), the second column time step 37 (T2) and the third column time step 49 (T3). The rows correspond to the ground truth manual segmentations and SIRT-FBP-MS-D-DIFF water classifications with standard, quasi-4D and ensemble training schemes of the MS-D network, respectively.
